# Storing paediatric genomic data for sequential interrogation across the lifespan

**DOI:** 10.1136/jme-2022-108471

**Published:** 2023-06-01

**Authors:** Christopher Gyngell, Fiona Lynch, Danya Vears, Hilary Bowman-Smart, Julian Savulescu, John Christodoulou

**Affiliations:** 1 Biomedical Ethics Research Group, Murdoch Children's Research Institute, Parkville, Victoria, Australia; 2 Department of Paediatrics, The University of Melbourne, Melbourne, Victoria, Australia; 3 Melbourne Law School, The University of Melbourne, Parkville, VIC, Australia; 4 University of South Australia, Adeliade, South Australia, Australia; 5 Faculty of Philosophy, University of Oxford, Oxford, UK; 6 Centre for Biomedical Ethics - Yong Loo Lin School of Medicine, National University of Singapore, Singapore; 7 Brain and Mitochondrial Research Group, Murdoch Children's Research Institute, Parkville, VIC, Australia

**Keywords:** Policy, Ethics- Medical, Databases- Genetic, Pediatrics

## Abstract

Genomic sequencing (GS) is increasingly used in paediatric medicine to aid in screening, research and treatment. Some health systems are trialling GS as a first-line test in newborn screening programmes. Questions about what to do with genomic data after it has been generated are becoming more pertinent. While other research has outlined the ethical reasons for storing deidentified genomic data to be used in research, the ethical case for storing data for future clinical use has not been explicated. In this paper, we examine the ethical case for storing genomic data with the intention of using it as a lifetime health resource. In this model, genomic data would be stored with the intention of reanalysis at certain points through one’s life. We argue this could benefit individuals and create an important public resource. However, several ethical challenges must first be met to achieve these benefits. We explore issues related to privacy, consent, justice and equality. We conclude by arguing that health systems should be moving towards futures that allow for the sequential interrogation of genomic data throughout the lifespan.

## Introduction

 Whole genome sequencing and whole exome sequencing (collectively genomic sequencing; GS) are increasingly being integrated into medicine. In recent years, governments have spent over US$4 billion to establish national genomics programmes, which have now collectively sequenced millions of individuals. This massive increase in genomic data is predicted to expand the opportunities and benefits of genomic medicine, increasing its utility in diagnosis, screening, prediction and research.

The last few years have seen GS increasingly used in paediatric settings. One example is the use of GS in critically ill children, where focusing on a rapid return of results helps diagnose genetic conditions in a matter of days.^1^ This testing has been touted as a ground-breaking application of genomic medicine, improving health outcomes for extremely unwell children in a cost-effective manner.^2^,^4^

Based on this success, there is a growing interest in using GS in healthy children to expand and enhance newborn screening (NBS) programmes.^4^ Currently, NBS programmes in developed countries typically follow a protocol of biochemical testing (commonly tandem mass spectrometry) as a tool to screen for a series of severe, early-onset, treatable conditions (such as inborn errors of metabolism or other inherited disorders), followed by GS as a second-tier confirmatory test.^5^,^7^ The use of GS as a first-tier test in NBS (in addition to biochemical testing) is starting to be investigated in several countries.^8 9^ Recently, the UK’s National Health Service announced its vision to sequence the genomes of up to 200 000 babies for up to 200 treatable, childhood-onset conditions.^8 10 11^ The National Institutes of Health in the USA also invested US$25 million into pilot programmes assessing the potential integration of GS into NBS,^9^ and the China Neonatal Genomes Project also aims to carry out GS of 100 000 neonates (both critically unwell and healthy) by 2025.^12^

While GS cannot replace biochemical testing for all conditions on NBS panels, it could improve the accuracy and scope of existing programmes. Several lethal childhood-onset conditions can only be detected through GS, including the neurodegenerative disorder Tay-Sachs disease, for which gene therapy is currently being developed.

One question emerging from the increased use of GS in children (including in NBS) is whether the data generated should be stored for future use. Recently, Johnson *et al* argued that there are strong moral reasons to store and share deidentified genomic data so that it can be used in research.^13^ Research using genomic data can lead to an improved understanding of diseases, novel treatments and produce substantial social benefits. In this paper, we explore a different moral reason to store genomic data: so that it can serve as a health resource for individuals throughout their lifespan.

There is a growing paradigm shift from viewing GS as a single diagnostic test to a lifetime health resource.^14 15^ A handful of authors have suggested models for the extended use of GS data from NBS.^14^,^17^ Most propose a two-stage approach to genomic NBS: stage 1: involving the implementation of GS into existing NBS programmes to improve their accuracy and identify other early-onset, treatable conditions; and stage 2: encompassing extended use of GS data for reanalysis at various time points following an individual’s medical needs and life stage.^14 15 17^ This approach of storing genomic NBS data and using it for sequential interrogation (SI) throughout an individual’s lifetime is the focus of this paper.

When considering whether to store paediatric genomic data for future use, many technical, practical, social and contextual issues must be considered. There might not be enough expertise, infrastructure and other resources to implement ongoing analysis of genomic data. Even if there were no material and resource constraints, there might be other barriers; an influx of genomic data might strain current health systems and lead to inefficiencies. For example, increased GS could divert funds and attention away from potentially more effective ways of improving health, such as improving living conditions.

Many national and international genomics programmes have been established to answer precisely these questions and find ways to implement genomics into our healthcare systems.^18^ In this paper, we assume that the practical, logistical and workforce challenges associated with storing and reusing genomic data can be met to highlight the underlying ethical considerations relevant to this issue. We aim to explicate ethical considerations relating to storing genomic data generated in genomic NBS programmes for the future medical benefit of the child. We draw on several different ethical perspectives to analyse this question, following a recognised method in bioethics.^19^

We first outline how an SI approach compares to practices of storing dried blood spots (DBS) from NBS, look at time points through the lifespan where the availability of genomic data could be useful, and compare SI to the alternative of sequencing on demand (SOD). We then look at broad ethical considerations that count in favour of genomic data storage and SI. These relate to an individual’s right to privacy, bodily sovereignty and the government’s general obligation to promote their citizens’ health and improve health equity. At the same time, storing paediatric genomic data for future clinical use raises novel ethical challenges that must be addressed. We discuss moral changes relating to false positives, variants of uncertain significance (VUS), data security, privacy and consent.

Like other medical interventions, ensuring the benefits of SI outweigh the costs will be essential. In our conclusion, we argue that governments should invest in systems that allow for the future benefits of SI to be realised while minimising the costs.

## Storing genomic data for SI

### From DBS to whole genome sequencing

Storing genomic data generated at birth might seem like a radical proposal. However, it can be seen as an extension of existing practices. Since the 1960s, newborns worldwide have routinely had a blood sample collected and stored, known as a DBS. The length of storage varies significantly across programmes, from just a few months to up to a lifetime. While stored DBS are mainly used for research, in some cases, they can also be accessed for the medical benefit of the child and their family.^20^

Storage of DBS has been controversial. There has been public opposition to the storage of DBS in some jurisdictions, leading to lawsuits and the forced destruction of DBS.^21^ Central to the public opposition to storing DBS is a lack of transparency about the storage and future use of DBS and a lack of control by individuals and their families.

Despite these controversies, studies of public attitudes toward DBS indicate support for storing and reanalysing DBS to provide medical benefits to the child or their family.^22^ Similarly, we may also expect genomic data storage for future medical use to have public acceptance. This is reflected in the recent public engagement work of Genomics England, which demonstrated widespread acceptance of collecting and storing genomic data from NBS, with the intention of reanalysis through the lifespan.^10^ Focus groups conducted with members of the Australian public found participants to be broadly supportive of storing genomic data for future medical use.^23^ Irrespective, it will be imperative for any programme storing NBS genomic data to learn from previous DBS programmes.

### Lifespan genomics

In several areas of medicine, reanalysing stored genomic data may benefit individuals and their families. These are outlined in table 1.

**Table 1:** Use of genomic data for SI throughout the lifetime

Application	Use	Benefit	Increased benefit of an SI approach
Childhood screening	GS during childhood.	Improve the accuracy of existing newborn screening and expand conditions detectable.^15 16^	Allow a staged approach to paediatric screening.
Childhood critical illness	Rapid GS for critically ill children.	Diagnosis can lead to life-saving treatment, reduce the need for invasive interventions and improve long-term outcomes.^49^	Remove the need to obtain consent, a new blood sample and sequence the DNA, therefore, reaching a diagnosis faster.
Reproductive carrier screening	GS to determine chance of having a child with genetic condition.^50^	Individuals can use this information for reproductive decision-making.	Reduce costs and increase the efficiency of reproductive carrier screening programmes.
Screening for chronic disease	GS for screening for chronic disease in adulthood.	Enhance or replace traditional screening techniques for chronic disease, improving risk prediction and empowering individuals to make informed diet and lifestyle choices to offset this risk.^44 51^	
Diagnosing and treating chronic disease	GS for diagnosis and management of chronic disease in adulthood.	Allow more tailored approaches to treatment of chronic disease, using known pharmacogenomic interactions.^52^	

GSgenomic sequencingSIsequential interrogation

There are currently mixed opinions about how generalisable results from genomic studies are, and whether published results replicate in diverse populations.^24^ As genomic databases grow and research improves, so will the utility of genomic data for screening, diagnosis, treatment and research.^24^ Because of this, we do not claim that each of these applications is ready for immediate clinical implementation. However, the widespread use of genomic technologies in each of the below areas is a plausible endpoint given the current trajectory of research and medicine.

### Ways of implementing an SI approach

There are many potential ways to organise genetic health services to allow for SI of stored genomic NBS data. Practices can vary regarding where genomic data is stored, the approach to consent, the interpretation of genomic data and what triggers reanalysis.

#### Storage of genomic data

Genomic data can be stored in a variety of ways. On one end of the spectrum, there are highly decentralised approaches, where individual genomes are stored on their own (perhaps on personal storage devices). In the middle of the spectrum, there are partially decentralised approaches, where genomic data from several hundreds or thousands of individuals is stored at local hospitals or health centres. At the far end of the spectrum, there are entirely centralised approaches where genomic data are stored in large national or international databases.

Each approach is compatible with SI of genomic data for medical benefit across the lifespan. But they will have different costs and efficiencies. For example, highly decentralised systems can pose less of a privacy risk, as it can be easier for people to maintain control over their data and be less of a target for hackers. However, these decentralised approaches may make data sharing more difficult and disproportionally benefit individuals with high levels of health literacy. At the other end of the spectrum, centralised approaches might be more efficient and make data sharing easier, but make it harder for individuals to maintain control over their data.

#### Consent for storage and reanalysis

We can also imagine a range of approaches to consent for storing genomic data from NBS, each with its costs and benefits. For DBS, some have taken the position that storage should be automatic as it is in the public interest.^21^ Similarly, we can imagine that automatic storing of genomic NBS data for future clinical use, without explicit consent, could be defended because it is both in the child’s best interest and serves the public interest. As discussed above, however, such an approach risks repeating the mistakes of previous DBS programmes where there was a large public backlash to storing children’s data without consent.

A potentially more permissive approach may be opt-out consent. Under this approach, genomic NBS data would be automatically stored, but families would be told of this and given the option to decline to have their child’s data stored or withdraw it later.

The final option is explicit (or opt-in) consent. However, using opt-in consent risks a reduced rate of uptake of such a programme, potentially limiting its wider public benefit. Within this approach, we can further distinguish between approaches that require only verbal/cursory consent, and those that require written consent.

#### Triggers for reanalysis

There are also different potential approaches to what triggers the interrogation of stored genomic data. One approach suggests family/patient-led requests could entirely drive reanalysis. For this approach, there would be no automatic analysis of genomic data, but it could be used as a resource in cases of illness if patients choose to make their data available.

Under a different approach, health systems could drive interrogation of stored genomic data by offering reanalysis at set time points. Some of these trigger points could be event-driven, like if individuals develop an acute illness. Other triggers could be age based; for example, when an individual reaches a certain age, they could be offered screening for diseases such as cancer.

In sum, we can imagine various ways health services may be organised to allow SI of stored genomic NBS data. In the next section, we outline broad ethical considerations that support storing data for SI, as well as ethical challenges and limitations that argue against this approach. But before we do, we need to look at a practical objection to storing and reanalysing genomic data: that SI approaches will not be as efficient as alternative SOD approaches.

### SI versus SOD

One practical objection to storing genomic data generated now is that sequencing technologies will likely improve over time, and resequencing will be required regardless of whether stored genomic data exists.^16^ Current approaches to GS use short-read sequencing techniques (such as those produced by market leader Illumina), which cannot accurately sequence some parts of the genome or detect several types of structural variants. Sequencing technologies that overcome these limitations are being developed and may one day be able to be of greater clinical utility than current technologies.^25^ When this happens, there will be a robust case for storing genomic NBS data for use across the lifespan.

The critical issue when considering the utility of storing data produced from today’s GS technologies is how accurate the data generated is for the regions of the genome that are sequenced. Short-read sequencing technology covers nearly all the genome’s protein-coding regions. There is currently relatively high confidence in the accuracy of this data, as evidenced by the rising use of GS in medicine. With the development of better technologies in the future, it could turn out that this confidence is misplaced and that current technologies are less accurate than once thought. Conversely, it could show that existing technologies are highly accurate for many parts of the genome. If the latter is true, it will imply that data produced using today’s sequencing technologies are still useful across many applications and can be a valuable lifetime health resource. Importantly, as more advanced sequencing technologies are developed, we will be able to determine how reliable old sequence data is and adjust practices accordingly.

Another objection to an SI approach is that it will eventually be cheaper to resequence a patient’s genome than to store and reanalyse genomic data generated in childhood. Sequencing costs have decreased dramatically in recent years, with the total cost per genome currently around US$500^26^ and predictions of US$100 genomes being made. While it is conceivable that the cost of sequencing will eventually be less expensive than storing a genome for a single year (currently about US$40^27^), the cost of storage will also likely reduce. While storage costs will be sensitive to context and may sometimes be prohibitive, recent evidence suggests that it is getting cheaper. One study estimated the cost of storing a single genome on a modern data server run by companies such as Amazon Web Service would be approximately US$14 over 10 years.^28^ Likewise, the data storage service provider for Genomics England, Weka, predicts the cost of storing one genome will be £2 per year by 2023.^29^

If data storage can be accessed cheaply, storing and reanalysing genomic data will be more cost-effective than resequencing an individual’s genome. Indeed, the more often someone’s genomic data is accessed over their lifetime, the more cost-effective it will be to use SI compared with an SOD approach.^14^ If, in the future, people’s genomic data are used multiple times over their lifespan, it seems unlikely that the cost-effectiveness of SOD will match storage and SI.

Moreover, SOD approaches require access to an individual’s body, which may be practically difficult in some cases. Some people have difficulties attending medical services in person. In some contexts, face-to-face contact with medical professionals is limited (such as in a pandemic or for patients who live far from health services).

While it is difficult to predict the future of genomic medicine, one plausible future is that storing genomic data will be more cost-effective and efficient than SOD approaches. It is, therefore, essential to consider the broader ethical implications of genomic NBS data storage and SI.

## Ethical considerations in favour of newborn genomic screening and SI

The shift from viewing GS as a single clinical test to a lifetime health resource has important ethical implications. These include considerations for an individual’s right to their genomic data, the potential benefits to public health, and improved equality in genomic medicine.

### The right to access one’s genomic data

It has recently been argued that patients and research participants have a moral—and sometimes a legal—right to genomic data generated from their cells in a research setting.^30^ On the one hand, this right is based on the right to privacy, which encompasses the right to control personal information that relates to oneself. This can be reasonably interpreted to infer that we should enjoy privacy over our genomic information. From a different perspective, a right to one’s genomic data extends from a right to bodily sovereignty; just as I should be the decision-maker concerning my body, I should be the decision-maker concerning my genomic data.

The claim to one’s genomic information generated in a clinical (rather than a research) setting may be even stronger. Not only do the arguments about privacy and bodily sovereignty apply, but there are additional considerations regarding a person’s right to their medical data. Patients are generally recognised to have an ethical and a legal right to access all medical data that doctors and health systems hold on them.^31^ Therefore, families participating in genomic NBS programmes may have the right to access the genomic data generated from their child’s cells. These suggested rights could be fulfilled by ensuring genomic data is stored securely (to protect privacy) and in an accessible format.

### Improved health outcomes

Another argument for storing genomic NBS data for future clinical use stems from the general obligation of governments to protect the health of their citizens. Promoting health can be seen as a vital role of governments as poor health can prevent people from participating in their society’s social, political and economic life. Furthermore, the International Covenant on Economic Social and Cultural Rights (ICESCR) recognises health as a fundamental human right, entailing ‘the right to the enjoyment of the highest attainable standard of physical and mental health’.^32^ This includes the right of citizens to access determinants of health, including health services that prevent and treat disease.

The ICESCR places a legal obligation on signatories to establish services that provide their citizens with the highest possible health standards. As outlined above, genomic data is already helping to improve disease prevention, diagnosis and treatment. If these trends continue and results replicate in diverse populations, we may reach a point where individuals benefit significantly from having their genomic data available for analysis throughout their lives. Given the strong obligation of governments to improve the health of their citizens, the potential of stored genomic data to achieve future health benefits provides strong reasons to develop systems that allow for such services.

### Improved equity in genomic medicine

A more speculative benefit of storing genomic NBS data for future use is that it will help remedy inequalities in genomic medicine. The growth of genomic medicine has been marked by injustice, where the benefits have been highly skewed towards those of northern European ancestry. In many health systems, a vicious cycle occurs, where pre-existing inequalities lead to minority ethnic groups being poorly represented in genomic databases, which in turn leads to the benefits of genomic research being skewed towards those already ‘best off’ in society, further entrenching these inequalities.

One way to break this cycle could be to generate large population-wide representative databases that can be used both for research and to inform clinical care. Implementing GS in NBS with the subsequent storage of genomic data could help achieve this. In the context of genomic analysis, accurate variant interpretation requires accurate population-level data. By incorporating genomic data from all newborns, population databases can better support variant curation and interpretation for majority and minority ethnic groups, providing a public benefit for all.

In addition to ethnic minorities, rural populations are also disadvantaged in genomic medicine. In the United States, the all-cause mortality rate is higher in rural areas than in urban areas, and this gap is increasing.^33^ Access to genetics services is also heavily restricted by location, with rural individuals less likely to receive necessary genetic testing.^34^ However, SI of genomic data generated through NBS could help address these disparities. Medical interventions that happen at birth have much greater coverage than interventions that occur later in life, with many NBS programmes achieving close to 99% coverage of the population.^6^ As such, NBS presents an ideal opportunity to generate a health resource that serves all members of a population. Furthermore, genomic data generated through NBS could be analysed remotely when needed, reducing the need for additional in-person appointments and disparities in access to genetic testing based on location. Likewise, it would be easier for members of rural and remote populations to participate in genomic research, further ensuring a more equitable division of precision medicine initiatives.

Another group that suffers a disadvantage in current health systems are those with rare diseases. While collectively common (with an estimated 1 in 100 people affected), rare diseases can affect as few as a handful of individuals worldwide. Increasing the volume of population-level genomic data available for research purposes is likely to increase the number of people with a rare disease that receive a genetic diagnosis, subsequently improving our understanding of these diseases’ genetic basis. Therefore, generating population-level genomic data could also benefit rare disease sufferers enormously.

The availability of millions of genomes would also significantly increase the power of genome-wide association studies, allowing the genetic basis of common polygenic diseases to be more readily understood. This could have implications for identifying at-risk populations or individuals and facilitate further research on treating common diseases.

## Ethical challenges in genomic NBS and SI

Above, we outlined ethical arguments in favour of storing genomic NBS data so that it can provide benefits throughout an individual’s life. These benefits must be balanced against the potential ethical risks of an SI approach and the incorporation of GS into NBS programmes more generally. This section discusses some of these risks and some strategies to mitigate them.

### False positives and uncertain findings

One concern with incorporating GS into NBS is that it will increase the number of false positives and uncertain results. As GS can be used to screen for hundreds of conditions simultaneously, it could increase the number who are falsely identified as being at an increased risk for a condition. Studies have shown that receiving a false positive through NBS increases parental stress and anxiety, at least in the short term.^35^ Parents whose children receive false positive results from NBS are significantly more likely to report needing extra parental support. Children who get false positive results are also more likely to require hospitalisation in the first six months of life, adding strain to healthcare resources.

Additionally, the uncertainty of genomic information could further increase parental anxiety. VUS occur when mutations are found in functional genes implicated in a disease but whose specific effects are unknown. If a VUS is pathogenic, then we can expect the overall impact of reporting it to be positive, as it can facilitate a diagnosis and access to treatments. However, if a reported VUS turns out to be benign, it may needlessly increase anxiety in parents, much like false positives. In addition to having harmful effects on parents, screening tests that resulted in people receiving many (ultimately benign) VUS could have detrimental effects on the health system. They could create ‘patients in waiting’, where a child is excessively monitored based on an uncertain finding.

While false positives and VUS are risks of incorporating GS into NBS, they are also risks of current biochemical NBS programmes. Because of this, guidelines that govern which conditions should be screened for already account for these risks. For example, the UK’s ‘criteria for appraising the viability, effectiveness and appropriateness of a screening programme’, states:

The benefit gained by individuals from the screening programme should outweigh any harms, for example from overdiagnosis, overtreatment, false positives, false reassurance, uncertain findings and complications*.*^36^

It will be essential if GS is incorporated into NBS—and genomic data subsequently stored and used in ongoing clinical care—that current ethical standards regarding false positives and uncertain results are upheld. This may mean that individuals are not screened for hundreds of conditions at birth but only those with favourable overall risk–benefit calculations. However, an advantage of an SI approach is that it allows for screening for other conditions to occur later, potentially triggered by risk factors such as personal and family history. Another advantage of this approach would be that it reduces the rates of incidental findings, where medical conditions are identified that were not the target of the original test. A moderate, measured approach to analysis, which takes account of contextual risk factors, could help maximise the benefits of GS while minimising the risk of inaccurate, uncertain or unexpected findings.

### Coercion and consent

In many current programmes, consent for NBS is cursory or implied. One criticism of the incorporation of GS into NBS programmes is that it will undermine this consent process and possibly the success of NBS programmes. These concerns will likely be amplified in programmes where genomic data is intended to be stored. Incorporating GS into NBS and its subsequent storage and reuse may put too many demands on the consent process and undermine the NBS programme overall.

Medical decisions about children can be ethically complex, as parents must consider a child’s current state and their long-term future. The decision to sequence a child’s genome might be especially difficult for parents, considering the complexity and volume of information required for informed consent. Furthermore, the perinatal period is a time of extremely heightened emotions, making the provision of informed consent for any test challenging.^37^ Despite these potential challenges, studies of parental attitudes towards expanding the number of conditions looked for in NBS have shown the clear majority are happy for their child to be screened without explicit consent.^38 39^

Irrespective of parents’ perspectives, an SI approach may mitigate some concerns about consent for genomic NBS. By enabling a staged approach to analysis, SI allows parents more time to weigh the benefits and costs of particular decisions. It would allow parents more time to ask and understand the answers to complex questions, particularly for those from diverse cultural and linguistic backgrounds.

This leaves open the question of what level of consent is required to store genomic data intended for future analysis. There will likely be diverse views on this question, with some calling for explicit written consent and others arguing that implied consent would be sufficient. One approach could be that parents are asked to provide consent for the analysis and storage of their child’s genomic data at birth to augment current NBS programmes and are then approached for subsequent consent for each additional analysis. However, achieving the greatest population-wide benefit from such programmes may require an implied or opt-out approach to genomic NBS data storage. Following this, parents could be provided sufficient support to make an informed decision about further stages of analysis, as well as given the option to discard any stored genomic data. This is an important area of future discussion and empirical exploration not included in this essay.

### Data governance and trust

Whether approaches such as SI are successful will, in large part, depend on trust. Recent experience with the COVID-19 pandemic has emphasised how important trust can be for the success of public health measures. Research across several countries during the pandemic found that trust in government was the biggest predictor of compliance with public health measures.^40^,^42^ Recent empirical research also suggests that people are reluctant to share genomic data if they have low trust in the organisations and individuals responsible for storage and sharing.^43^

A considerable influence on how people trust data collection and storage initiatives such as SI will depend on systems implemented for data governance and security. One critique of an approach such as SI is that the data stored would not be secure, which could reduce trust in genomic NBS programmes more broadly. Participants in the recent Genomics England public dialogue on genomic NBS suggested that data security measures were crucial ‘to prevent data loss through hacking or human error and to build trust in the programme’.^44^ High-profile data breaches, such as the 2017 Ransomware attack, have been cited as factors that might reduce public trust in programmes such as SI.^45^ Robust systems for data security—which can protect stored genomic data from hackers and coordinated cyber attacks—will be essential for initiatives like SI to be successful.

The need to develop systems of data governance that build trust in the community and efficiently deliver positive health outcomes is a crucial challenge for approaches such as SI and genomic medicine more generally. Apart from establishing robust data security measures, how can an SI approach be designed to elicit community trust? One suggestion is that community members will likely have confidence in systems when they have some control over who has access to their genomic data and what it is used for.^44^ If genomic databases can be accessed by insurers, employers or law enforcement, this may significantly reduce trust.

### Protecting privacy

To be used in an individual’s future clinical care, genomic information would have to be stored with personal and other phenotypic information. This creates a much greater privacy risk than the storage of deidentified data.

Privacy has long been a concern of GS.^46^ However, the relationship between GS and privacy is highly complex. While genetic privacy is often named as a concern of large-scale GS, this is more frequently discussed in the context of publicly available genomic data.^47^

There are several ways to conceive of privacy, each with different implications for genomic NBS. For example, in some concepts of privacy, we enjoy privacy over our information provided we retain control of that information:

Privacy is not simply an absence of information about us in the minds of others; rather it is the control we have over information about ourselves…The person who enjoys privacy is able to grant or deny access to others.^48^

With this view, generating personal information about someone does not necessarily breach privacy, whereas sharing this information without their permission would. Privacy considerations do not necessarily count against storing genomic NBS data for future medical use if individuals and families maintain control over the data.

Another view of privacy is that of a cluster concept consisting of rights over one’s body, personal information and property^47^. For example, we have the right to confidentiality over our personal information, and if this information is shared without our consent, our rights are violated. We also have the right not to be harmed by others. If the release of our personal information harms us in some way, our rights have been violated. Within this view, having one’s genome sequenced at birth is not necessarily an invasion of privacy, provided the information is used in ways that do not violate our right to confidentiality or our right not to be harmed.

Generating and storing genomic data need not breach one’s right to privacy. Instead, privacy can be upheld by careful management of storage, access and use of such information. Privacy could also be protected by allowing individuals control over how their genomic data is used and allowing them to delete their data if desired.

## Conclusion

As genomic NBS is being trialled around the world, the issue of how to handle the large amounts of genomic data produced is becoming increasingly significant. Whether we should store genomic data generated in NBS programmes depends on a wide range of factors, including infrastructure, workforce capacity and evidence of cost-effectiveness. Sitting alongside these practical considerations are ethical ones regarding the rights of individuals, families and the obligations of governments and healthcare systems. Families can be seen to have a right to their child’s genomic data, grounded in the rights of privacy and bodily sovereignty. Furthermore, governments have both legal and ethical obligations to grant access to the determinants of health for their citizens, which can be reasonably argued to extend to access to their own genomic data. Additionally, equity has long been an issue in the delivery of genomic medicine, and the collection of population-wide genomic data at birth could help to reduce these disparities.

Storing genomic NBS data also introduces several ethical risks. We suggest these can be seen as obstacles to be overcome rather than concrete barriers. A key lesson from the storage of DBS is that trust and transparency are critical factors for the long-term success of these programmes. Building trust will require forms of governance that allow people control over their data, which will also help mitigate privacy risks. The increased use of genomic data in medicine may result in more false positives, overdiagnosis and uncertain findings. Still, these risks can potentially be mitigated through targeted analyses that consider individual background risk. As more NBS programmes generate GS data, thought should be given to ways this valuable data can be stored and utilised rather than discarded.

## Data Availability

No data are available.
